# Inflammatory Signals Across the Spectrum: A Detailed Exploration of Acute Appendicitis Stages According to EAES 2015 Guidelines

**DOI:** 10.3390/diagnostics14202335

**Published:** 2024-10-21

**Authors:** Maximilian Dölling, Mihailo Andric, Mirhasan Rahimli, Michael Klös, Jonas Pachmann, Jessica Stockheim, Sara Al-Madhi, Cora Wex, Ulf D. Kahlert, Martin Herrmann, Aristotelis Perrakis, Roland S. Croner

**Affiliations:** 1 University Clinic for General-, Visceral-, Vascular and Transplantation Surgery, Faculty of Medicine, Otto-Von-Guericke University, 39120 Magdeburg, Germanyulf.kahlert@med.ovgu.de (U.D.K.); aristotelis.perrakis@med.ovgu.de (A.P.); roland.croner@med.ovgu.de (R.S.C.); 2 Molecular and Experimental Surgery, Department of General-, Visceral-, Vascular and Transplant Surgery, Faculty of Medicine and University Hospital Magdeburg, Otto-Von-Guericke University, 39120 Magdeburg, Germany; 3 Department of Pediatric Surgery, University Medical Center Mannheim, University of Heidelberg, 68167 Mannheim, Germany; martin.herrmann@uk-erlangen.de; 4 Department of Internal Medicine 3—Rheumatology and Immunology, Uniklinikum Erlangen, Friedrich Alexander University Erlangen-Nürnberg (FAU), 91054 Erlangen, Germany; 5 Deutsches Zentrum für Immuntherapie (DZI), Uniklinikum Erlangen, Friedrich Alexander University Erlangen-Nürnberg (FAU), 91054 Erlangen, Germany; 6 Department of General, Minimally-Invasive Surgery and Surgical Oncology, Center for Hepatobiliary and Colorectal Surgery, Iatriko Medical Center, 15125 Athens, Greece

**Keywords:** acute appendicitis, inflammatory markers, c-reactive protein, leucocyte count, EAES 2015

## Abstract

**Background**: In this retrospective study, we evaluate the diagnostic utility of C-reactive protein (CRP) and leucocyte count within the EAES 2015 guidelines for acute appendicitis (AA) in differentiating uncomplicated (UAA) from complicated AA (CAA). **Methods:** Conducted at a tertiary care center in Germany, the study included 285 patients over 18 years who were diagnosed with AA from January 2019 to December 2021. Patient data included demographics, inflammatory markers, and postoperative outcomes. **Results:** CRP levels (Md: 60.2 mg/dL vs. 10.5 mg/dL; *p* < 0.001) and leucocyte count (Md: 14.4 Gpt/L vs. 13.1 Gpt/L; *p* = 0.016) were higher in CAA. CRP had a medium diagnostic value for detecting CAA (AUC = 0.79), with a cutoff at 44.3 mg/L, making it more likely to develop CAA. Leucocyte count showed low predictive value for CAA (AUC = 0.59). CRP ≥ 44.3 mg/L was associated with a higher risk of postoperative complications (OR: 2.9; *p* = 0.002) and prolonged hospitalization (OR: 3.5; *p* < 0.001). **Conclusions:** CRP, within the context of the EAES classification, presents as a valuable diagnostic marker to distinguish CAA from UAA, with a higher risk of postoperative complications and hospitalization. Leucocyte count showed low diagnostic value for the identification of CAA.

## 1. Introduction

Acute appendicitis (AA) is a common surgical emergency worldwide, with an annual incidence rate of 228 cases per 100,000 individuals [1]. The lifetime probability of developing AA is estimated at 8.6% for males and 6.7% for females [2]. This showcases peak incidence rates among adolescents aged 10–14 years for males and 15–19 years for females [3]. Large clinical trials suggest non-operative treatment (NOT) by the application of antibiotics as an alternative for cases presenting with a mild clinical progression termed “simple” or “uncomplicated” acute appendicitis [4,5]. The identification of patients with uncomplicated appendicitis that would benefit from NOT is a challenge in the clinical setting [6,7]. On the one hand this is because the diagnosis of AA relies on a combination of patient history, clinical presentation, results of physical examination, and imaging [8]. It is aided by laboratory markers such as C-reactive protein (CRP), leucocyte count, and differential blood counts [9,10,11]. Despite improvements in imaging-based diagnostics due to widely available abdominal ultrasound and CT-scan, the diagnosis and identification of subclasses of AA remains difficult and leads to a relevant rate of perforation (22–62%) and, finally, to a high rate of negative appendectomies [12,13]. For this reason, efforts have been made to improve diagnostics by the implementation of new laboratory markers to aid the identification of AA [14]. These include CRP-to-leucocyte ratio, leucocyte-to-lymphocyte ratio, procalcitonin, hyperbilirubinemia, and bilirubin-to-leucocyte ratio, to mention some [15,16,17,18,19]. Additionally, scoring systems such as the Alvarado score or Appendicitis Inflammatory Response (AIR) score were developed, which include patient characteristics, comorbidities, and symptoms to maximize diagnostic accuracy [20,21]. On the other hand, a globally heterogeneous classification systems of simple and advanced forms of AA based on clinical and pathological findings make it difficult to compare therapeutic strategies [22]. Given the global diversity in classification and treatment, conferences on AA were convened, such as by the European Association of Endoscopic Surgery (EAES) in 2015, and in Jerusalem 2016 and 2020 (WSES) [23,24,25]. The aim was to establish standards for diagnostic criteria and management of AA [26]. In accordance with the guidelines set forth by the EAES, individuals diagnosed with AA are stratified into uncomplicated (UAA) and complicated AA (CAA) [26]. UAA is defined by simple inflammation of the appendix vermiformis without surrounding reaction or appendix wall defect. CAA is defined by free abdominal fluid, presence of surrounding phlegmon, intraabdominal abscesses, signs of gangrene or perforation [26]. CAA is diagnosed if at least one of the above criteria is met, leading to the decision for appendectomy [26].

Previous studies aimed to evaluate the diagnostic utility of predetermined thresholds for CRP and leucocyte count in the diagnosis of advanced forms of AA, which are typically gangrenous and perforated appendicitis [27,28,29,30,31,32,33,34,35]. Nonetheless, these studies were heterogeneous in the classification of subgroups as UAA or CAA and did not utilize the updates of the EAES classification system, which also categorizes phlegmonous acute appendicitis as CAA [17]. To address this deficiency and to support the standardization of diagnostics and treatment of AA, the aim of our study was to investigate CRP and leucocyte count within the EAES classification guidelines for AA.

## 2. Materials and Methods

### 2.1. Study Design

This study is a retrospective study that was planned and conducted in accordance with the rules of “good clinical practice” and the Declaration of Helsinki. An institutional review board approved the protocol (EA 173/22). All participants were given patient information, and they provided written informed consent. The authors vouch for the completeness and accuracy of the data.

### 2.2. Patients

All patients over the age of 18 years admitted to the University Hospital Magdeburg, Saxony-Anhalt, Germany, diagnosed with “acute appendicitis”, who provided informed consent and underwent surgical removal of the vermiform appendix from January 2019 until December 2021, were included in the study. Patients with acute appendicitis who were not operated on or who had missing laboratory parameters were excluded from the study.

### 2.3. Outcomes

The primary outcome was the intraoperative stage of disease. Secondary outcomes were subgroups of AA, morbidity, and hospitalization.

### 2.4. Definitions and Data Collection

In adherence to the Sex and Gender Equity in Research (SAGER) Guidelines, we reported differences in gender by self-reporting of sex assigned at birth. According to the EAES (European Association of Endoscopic Surgery) guidelines of 2015, we defined UAA as catarrhalic AA and CAA as phlegmonous, gangrenous, or perforated AA, including perityphilitic abscess based on intraoperative diagnosis.

### 2.5. Statistical Analysis

Data collection was performed using Microsoft Excel (v16.79.1, Microsoft, Redmond, WA, USA), and the statistical analysis was conducted with the use of STATA (v17.0, StataCorp., College Station, TX, USA) and SPSS (v28, IBM, Armonk, NY, USA). Graphical representations were generated using GraphPad PRISM (v9, GraphPad Software, Inc., Boston, MA, USA). To ensure the reproducibility of results, a comprehensive log of the statistical analysis was maintained. Analysis of non-parametric variables involved the use of contingency tables, with chi-squared test statistics for assessment. The Shapiro-Wilk tests, along with the evaluation of histograms, were employed to ascertain the normal distribution of continuous variables before their testing. In case of non-normal distribution, the homogeneity of variance was assessed using F-tests or Levene’s robust test. For distributions that were skewed, the Wilcoxon-Mann-Whitney tests were utilized. The presentation of qualitative and quantitative data was achieved through the use of case numbers and percentages, means/medians (Md), standard deviations/interquartile ranges (IQR), and odds ratios (OR), respectively. A significance level was established for all statistical tests, setting a critical *p*-value at 0.05 with two-sided testing.

ROC regression analyses were conducted to depict the models, and the area under the curves (AUC) was calculated. The optimal cutoff value for CRP, in terms of sensitivity and specificity, was determined by calculating the Youden index.

## 3. Results

### 3.1. Population and Primary Outcome

Table 1 displays basic patient characteristics of the study population. From January 2019 until December 2021, a total of 285 patients who underwent surgery for acute appendicitis (AA) were included in the study. According to the EAES guidelines from 2015, 193 (67.7%) and 92 (32.3%) of patients were classified as CAA and UAA, respectively. Analyzing the advanced stages, we observed phlegmonous (*n* = 99; 34.7%), gangrenous (*n* = 31; 10.9%), and perforated AA (*n* = 49; 17.2%). Intraperitoneal abscesses were found in 14 patients (4.9%). A total of 39 patients developed postoperative surgical complications (13.7%), with 13 patients requiring surgical or radiological intervention (4.6%).

### 3.2. CRP and Leucocyte Count Are Elevated in CAA, and CRP Increases with Stages of AA According to the EAES Guidelines from 2015

**Figure 1:**
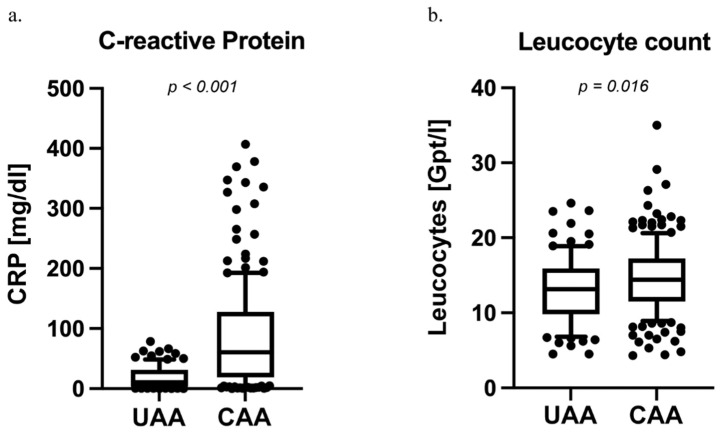
Preoperative CRP and leucocyte levels in patients with uncomplicated (UAA) and complicated acute appendicitis (CAA). (**a**) Among 285 patients with acute appendicitis, those with complicated acute appendicitis (CAA), including cases of phlegmonous appendicitis, had significantly higher preoperative CRP levels compared to those with uncomplicated acute appendicitis (UAA) (Median CAA = 60.2, 19.8; 124.9 vs. Median UAA = 10.5, 3.7; 31.2; *p* < 0.001). (**b**) Leucocyte counts were also elevated in patients with intraoperatively confirmed CAA in comparison to UAA (Median CAA = 14.4, 11.5; 17.2 vs. Median UAA = 13.1, 9.9; 15.9; *p* = 0.016).

At the time of hospital admission, we assessed the inflammatory markers CRP and leucocyte count (see Figure 1a). Patients with intraoperatively diagnosed CAA (Md = 60.2, 19.8; 124.9) were found to have higher CRP levels compared to patients with UAA (Md = 10.5, 3.7; 31.2; *p* < 0.001). The leucocyte count was also increased in CAA (Md = 14.4, 11.5; 17.2) compared to UAA (Md = 13.1, 9.9; 15.9; *p* = 0.016). (see Figure 1b).

**Figure 2:**
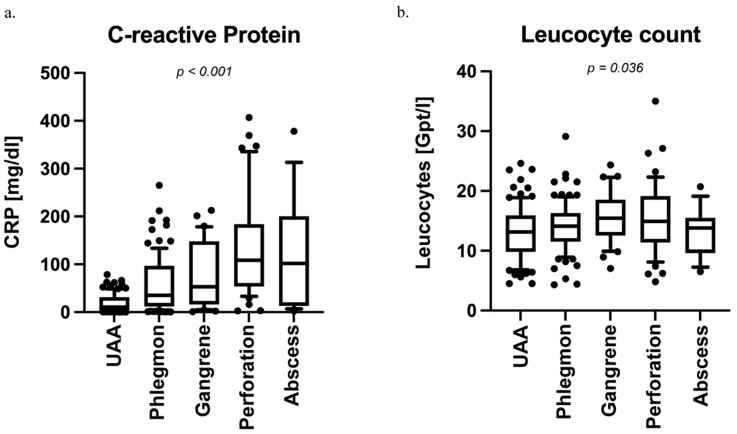
Subgroup analysis of acute appendicitis (AA) stages and associated inflammatory markers. (**a**) Intraoperatively diagnosed AA stages showed a significant increase in preoperative CRP levels corresponding to disease severity. CRP levels were significantly elevated in patients with phlegmonous AA (Median = 35.2, 11.9; 96.7), gangrenous AA (Median = 53.2; 15.9; 148.3), perforated AA (Median = 108.4; 54.1; 179.2), and abscesses (Median = 101.8; 13.6; 192.9) compared to those with uncomplicated AA (UAA) (ANOVA, F = 30.19, *p* < 0.001). Post-hoc analysis indicated significant differences between perityphlitic abscesses (*p* = 0.020) and perforated AA (*p* < 0.001) when compared to phlegmonous AA. A significant difference was also observed between perforated and gangrenous AA (*p* = 0.002), while no significant differences were found between gangrenous and phlegmonous AA (*p* = 0.733) or between perforated AA and abscesses (*p* = 1.000). (**b**) Leucocyte counts varied across AA stages (F = 3.14; *p* = 0.036), but post-hoc analysis identified a significant difference only between perforated AA and UAA (*p* = 0.030).

We performed a one-way ANOVA to analyze the subgroups of patients with CAA and showed rising CRP levels of clinical relevance with advanced clinical stages: phlegmonous (Md = 35.2, 11.9; 96.7), gangrenous (Md = 53.2; 15.9; 148.3), and perforated AA (Md = 108.4; 54.1; 179.2) and abscesses (Md = 101.8; 13.6; 192.9) (F = 30.19, *p* < 0.001; Figure 2a). The post-hoc analysis revealed differences between perityphilitic abscesses (*p* = 0.020) and perforated AA (*p* < 0.001) from phlegmonous AA. The perforated AA differed significantly from the gangrenous form (*p* = 0.002). Interestingly, no difference could be detected between gangrenous and phlegmonous AA (*p* = 0.733) and between perforated AA and abscesses (*p* = 1.000). Leucocyte count also differed between stages of AA (F = 3.14; *p* = 0.036). However, ad-hoc analysis revealed only a significant difference between perforated AA and UAA (*p* = 0.030 Figure 2b).

### 3.3. ROC-Analysis Revealed Medium Diagnostic Value of CRP and No Diagnostic Value for Leucocyte Count for CAA

**Figure 3:**
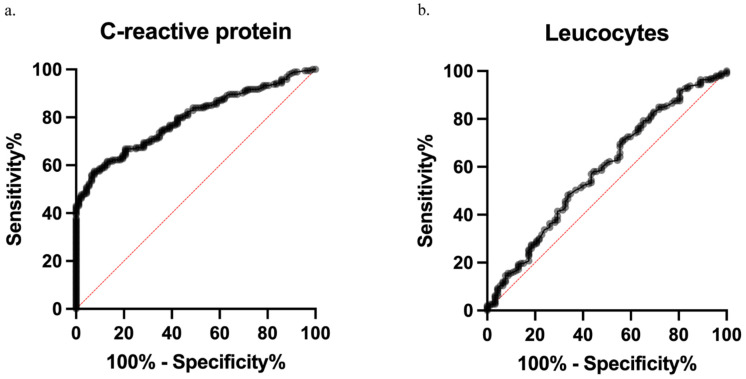
Receiver operating characteristic (ROC) analysis of inflammatory markers in the standard diagnostics of acute appendicitis (AA). (**a**) The ROC analysis demonstrates that CRP has a moderate predictive value for complicated acute appendicitis (CAA), with an area under the curve (AUC) of 0.79. The red line represents an AUC of 0.5, indicating no diagnostic value. Using the Youden index, the optimal CRP cutoff was determined to be 44.3 mg/dL, with a sensitivity of 0.62 and a specificity of 0.87 (AUC cutpoint = 0.74). (**b**) A separate ROC analysis indicated that leucocytes have a low predictive value for CAA, with an AUC of 0.59.

To investigate the value of CRP and leucocyte count as diagnostic tools for CAA, we performed a ROC analysis to reveal the medium diagnostic value of CRP (AUC = 0.79). The optimal cutoff of CRP was 44.3 mg/L, with a sensitivity of 0.62 and a specificity of 0.87 (AUC_cutpoint_ = 0.74; see Figure 3a). As expected, patients with CRP values ≥ 44.3 mg/dL (*n* = 119) were significantly more likely to have CAA diagnosed intraoperatively. Only 13 patients with CRP values ≥ 44.3 mg/dL showed UAA intraoperatively. With CRP < 44.3 mg/dL, UAA dominated (*n*_UAA_ = 79; vs. *n*_CAA_ = 74) (*p* < 0.001). Leucocyte count showed low diagnostic value with an AUC of 0.59 (see Figure 3b). For this reason, leucocyte count was excluded from further analyses.

### 3.4. Patients with CAA Carry a Higher Risk of Postoperative Complications

**Figure 4:**
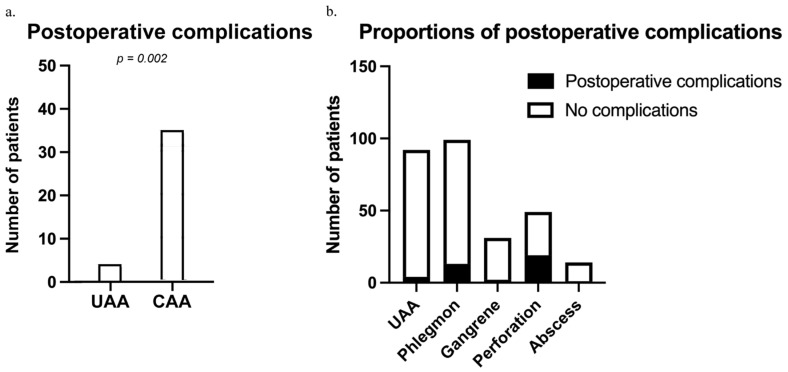
Comparison of postoperative complications between patients with uncomplicated (UAA) and complicated (CAA) acute appendicitis, as well as subgroups of acute appendicitis (AA). (**a**) In this study, 39 out of 285 patients (13.7%) experienced postoperative complications. Patients with complicated acute appendicitis (CAA) (*n* = 35) had a significantly higher incidence of postoperative complications compared to those with uncomplicated acute appendicitis (UAA) (*n* = 4; *p* = 0.002). (**b**) Subgroup analysis (ANOVA) showed that 4.13% of patients with UAA (*n* = 4) developed complications. Patients with phlegmonous appendicitis had a complication rate of 13.13% (*n* = 13; *p* = 0.041). There were no significant differences in complication rates between UAA and patients with gangrenous AA (6.45%, *n* = 2; OR: 1.51; *p* = 0.6412) or those with abscesses (7.14%, *n* = 1; OR: 1.69; *p* = 0.5147). However, patients with perforated AA had a significantly higher rate of postoperative complications, occurring in 38.78% of cases (*n* = 19; OR: 13.93; *p* < 0.001).

The clinical impact of the different stages of AA was analyzed using risk analysis for postoperative morbidity, including the development of minor (Clavien–Dindo classification < III) and major postoperative complications requiring surgical or non-surgical interventions (Clavien–Dindo-classification ≥ IIIa). Postoperative complications (N = 39) were more frequent in patients with CAA (*n* = 35) compared to patients with UAA (*n* = 4) (*p* = 0.002; see Figure 4a). The odds of developing postoperative complications in patients with CAA were 4.87 compared to UAA (OR = 4.87; *p* = 0.001). There was no difference in patients with UAA and CAA in the necessity for interventions when developing postoperative complications (Clavien–Dindo ≥ IIIa; OR = 2.98; *p* = 0.239).

Subgroup analysis revealed the varying risks depending on the intraoperative stage of disease to develop postoperative complications (see Figure 4b). Patients with phlegmonous AA developed postoperative complications in 13.13% (*n* = 13) of postoperative complications compared to 4.13% in UAA patients (*n* = 4; OR: 3.32; *p* = 0.041). Complications included postoperative intraabdominal abscesses as well as ileus and cecal insufficiency. Gangrenous AA and abscesses showed no relevant differences in complication rates compared to UAA, with a risk of 6.45% (*n* = 2; OR: 1.51; *p* = 0.6412) and 7.14% (*n* = 1; OR: 1.69; *p* = 0.5147), respectively. Patients with perforated AA developed postoperative complications in a total of 38.78% of cases (*n* = 19; OR: 13.93; *p* < 0.001).

### 3.5. Hospitalization Is Prolonged in Patients with Complicated Acute Appendicitis

**Figure 5:**
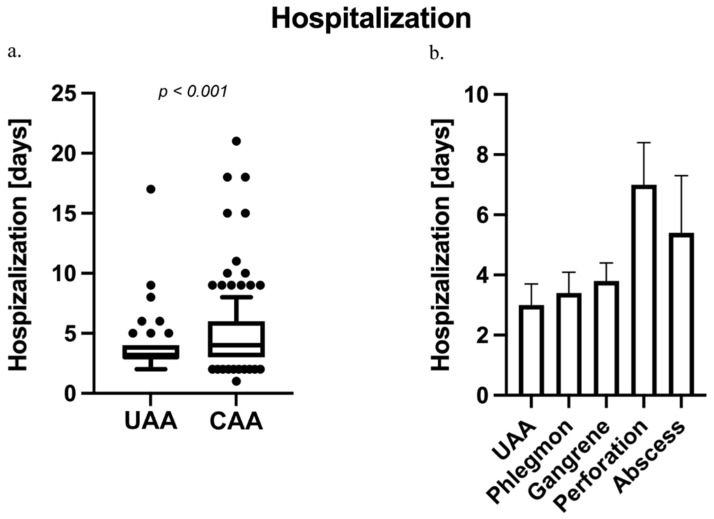
Duration of hospitalization in patients with acute appendicitis (AA). (**a**) Patients with complicated acute appendicitis (CAA) had a longer median hospital stay (Md = 4.0; 3.0; 6) compared to those with uncomplicated acute appendicitis (UAA) (Md = 3.0; 2.7; 3.6; *p* < 0.001). (**b**) In the CAA subgroup analysis, patients with perforated appendicitis had significantly extended hospitalizations (Md = 7.0; 5.2; 8.4) compared to patients with UAA as well as phlegmonous (Md = 3.4; 3.1; 4.1; *p* < 0.001) and gangrenous appendicitis (Md = 3.8; 3.3; 4.4; *p* < 0.001) (F = 19.72; *p* < 0.001). Additionally, patients with abscesses (Md = 5.4; 4.4; 7.3) also had a longer hospital stay than those with UAA (*p* = 0.006).

Besides the rate of postoperative complications, we analyzed the duration of hospitalization in days (see Figure 5a). Patients with CAA (Md = 4.0; 3.0; 6; *p* < 0.001) were hospitalized for a significantly longer duration compared to patients with UAA (Md = 3.0; 2.7; 3.6). Subgroups of patients with CAA revealed prolonged hospitalization in patients with perforation (Md = 7.0; 5.2; 8.4; *p* < 0.001) compared to patients with UAA, phlegmonous (Md = 3.4; 3.1; 4.1; *p* < 0.001), and gangrenous AA (Md = 3.8; 3.3; 4.4; *p* < 0.001), respectively (F = 19.72; *p* < 0.001). Additionally, patients with abscesses (Md = 5.4; 4.4; 7.3; *p* = 0.006) had a longer hospitalization time than patients with UAA (see Figure 5b).

### 3.6. Increased CRP Is Associated with a Higher Risk of Postoperative Complications and Prolonged Hospitalization

**Figure 6:**
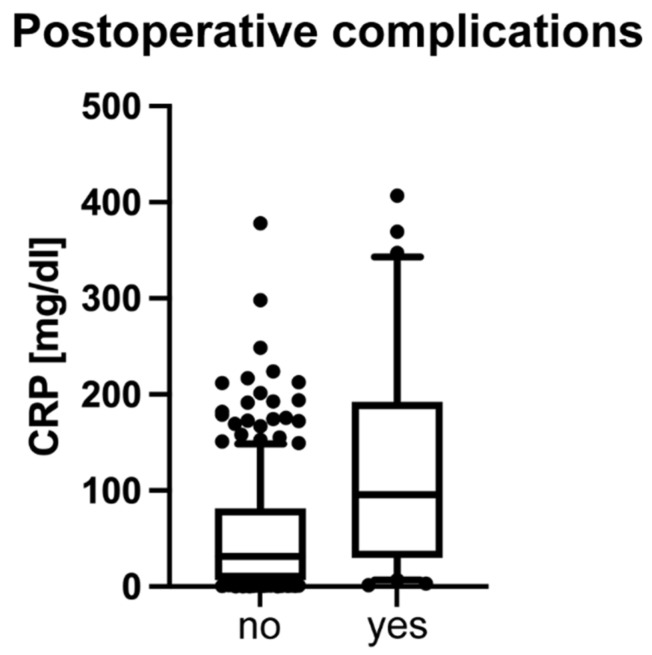
Preoperative CRP levels in patients with postoperative complications. Patients who experienced postoperative complications had significantly higher preoperative CRP levels (Median = 95.8; 29.9; 192.2; z = −3.980) compared to those who did not develop complications (Median = 54.3; 6.8; 80.6; *p* < 0.001).

Patients with CAA and increased preoperative CRP levels developed postoperative complications more often compared to patients with UAA (Md = 95.8; 29.9; 192.2; z = −3.980; *p* < 0.001 and Md = 54.3; 6.8; 80.6; see Figure 6). By using the cutoff CRP ≥ 44.3 mg/L for CAA, we analyzed the odds for postoperative complications. A contingency table revealed an odds ratio of 2.9 (*p* = 0.002; see Table 2) for complications when CRP was ≥44.3 mg/L. Complications were conditions requiring interventions according to the Clavien–Dindo classification: wound infection (*n* = 4), intraabdominal abscess (*n* = 1), appendix stump leakage (*n* = 2), trocar hernia (*n* = 1), intraabdominal bleeding (*n* = 1), respiratory insufficiency (*n* = 2), and ileus (*n* = 1). In addition, patients with CRP ≥ 44.3 mg/L were hospitalized for longer (Md = 4.3; 3.3; 6.2; *p* < 0.001) than those with CRP < 44.3 mg/L (Md = 3.4; 3.1; 4.3) and showed an increased risk for longer hospital stay (OR: 3.5; *p* < 0.001; see Table 3).

## 4. Discussion

In previous studies, CRP levels and leucocyte count increased with pathological and clinical stages of disease in children and adults [36,37]. However, classification and subgroup analysis in the literature were not standardized, and the lack of validated systems for the classification of acute appendicitis leads to controversy [22,29]. For instance, phlegmonous appendicitis was heterogeneously classified either as uncomplicated acute appendicitis (UAA) or, less commonly, as complicated acute appendicitis (CAA), according to the updated literature [22]. Therefore, an EAES consensus paper standardized the classification of AA into UAA and CAA, graded by clinical features of different stages of disease [17]. CAA applies to patients with either an inflamed appendix vermiformis with peri-appendicular phlegmon, purulent free fluid, or a gangrenous inflamed appendix with or without perforation, and intra-abdominal abscesses [17]. In the absence of these factors, AA was classified as UAA [38]. If a CAA is suspected, patients are mostly recommended to receive appendectomy [38,39]. Therefore, correct diagnosis and, if possible, pre-therapeutical differentiation of UAA and CAA is relevant for choosing adequate treatment.

In clinical practice, CRP and leucocyte count are commonly used laboratory markers for the diagnosis of suspected acute appendicitis [17]. Single inflammatory markers are not reliable, leading to the inclusion of further factors reflected by various clinical scores for the diagnosis of AA [17,20,40,41,42]. Interestingly, meta-analysis of the diagnostic value of inflammatory markers for suspected acute appendicitis revealed that CRP is the most important inflammatory marker, followed by leucocyte count and procalcitonin [17,43]. In those studies, CRP was elevated in advanced stages of AA with different cutoff values most likely due to the heterogeneity of classification systems applied. Also, these studies yielded only medium accuracy of 0.75 for CRP and 0.72 for leucocyte count in the diagnosis of AA [17,43]. Currently, little is known about CRP levels and leucocyte count based on the classification system established by the EAES 2015 guidelines for the differentiation between UAA and CAA and their implications for complication rate and hospitalization.

The study classified AA according to the EAES guidelines as either CAA or UAA, with CAA observed in 67.7% of the cases. Inflammatory markers, specifically CRP levels and leucocyte count, were significantly elevated in patients with CAA compared to those with UAA. A similar observation has been reported in previous studies. Notably, although cases of phlegmonous appendicitis were classified as uncomplicated acute appendicitis (UAA) in earlier studies, and in our study were grouped with complicated acute appendicitis (CAA) according to the 2015 EAES guidelines, the significantly higher leucocyte and C-reactive protein (CRP) levels observed in CAA cases compared to UAA remain unaffected by this reclassification. Similar to previous literature, in our study, CRP has a moderate diagnostic value for the identification of CAA, while the diagnostic value of leucocyte count was low, indicating its minor clinical use.

This study also explored the risk of postoperative complications, revealing a significantly higher incidence in patients with CAA. Correlating preoperative CRP levels with outcomes indicated that higher CRP levels were associated with an increased risk of postoperative complications and prolonged hospitalization. Specifically, patients with perforated appendicitis showed a remarkably higher rate of complications and hospitalization.

The medium accuracy of CRP for the diagnosis of CAA in our data indeed necessitates a more comprehensive diagnostic approach. Incorporating various further diagnostic criteria and tools to enhance accuracy and reduce the risk of misdiagnosis is also required within the EAES classification system. Efforts were made to identify new methods for the stratification of AA, such as AI-based diagnosis, neutrophil percentage, procalcitonin, IL-1, IL-6, bilirubin, urinary 5-HIAA, pentraxin-3, ischemia-modified albumin, myeloid-related protein 8/14 complex (MRP 8/14), mean platelet volume, citrullinated histone H3 (citH3), and myeloperoxidase (MPO). However, no such biomarker has yet been established in clinical practice [44,45,46,47,48,49,50,51,52].

## 5. Conclusions

CRP remains the key marker for diagnosing complicated appendicitis (CAA); however, its moderate diagnostic accuracy in detecting CAA suggests the need for new complementary biomarkers. While leucocyte count is reliable, identifying novel molecular or inflammatory markers could enhance diagnostic precision. Future research should focus on finding and validating these markers through clinical trials. Until then, diagnosis should rely on a combination of CRP, scoring systems, and imaging techniques, such as ultrasound or CT, supported by clinical evaluation.

## Figures and Tables

**Table 1:** Patient characteristics.

Parameters		*n* (%) or Mean (SD)/Median (IQR)
Gender	Male	163 (57.2)
	Female	122 (42.8)
Age [yrs]		41 (27; 58)
BMI * [kg/m^2^]	<18.5	5 (1.8)
	18.5–24.9	108 (37.9)
	25.0–29.9	104 (36.5)
	30.0–34.9	42 (14.7)
	35.0–39.9	13 (4.6)
	>40.0	13 (4.6)
Diabetes	Yes	20 (7.0)
	No	265 (93.0)
ASA ** score	I	89 (31.2)
	II	153 (53.7)
	≥III	43 (15.1)
Inflammatory markers		
Leucocyte count [Gpt/L]		14.0 (10.9; 16.7)
C-reactive protein [mg/L]		35.9 (8.9; 96.7)
Appendicitis	Complicated	193 (67.7)
Incl. subgroups	Phlegmon	99 (34.7)
	Gangrene	31 (10.9)
	Perforation	49 (17.2)
	Abscess	14 (4.9)
	Uncomplicated	92 (32.3)
Postop. complications	Total	39 (13.7)
	CD *** < III A	26 (9.1)
	CD *** ≥ III A	13 (4.6)

* Body mass index ** American Society of Anesthesiologists *** Clavien–Dindo classification.

**Table 2:** Dependency of odds for complications and CRP.

	CRP ≥ 44.3 mg/L	CRP < 44.3 mg/L	Total	Proportion Exposed
Complications	25	14	39	0.641
No Complications	94	152	246	0.382
Total	119	166	285	0.417
	OR 2.89		*p* = 0.002	

**Table 3:** Dependency of odds for hospitalization in days and CRP.

	CRP ≥ 44.3 mg/L	CRP < 44.3 mg/L	Total	Proportion Exposed
≥4.3 days	51	63	114	0.447
<4.3 days	31	134	165	0.188
Total	82	197	279	0.294
	OR 3.50		*p* < 0.001	

## Data Availability

All data generated or analyzed during this study are included in this published article.
